# The hemodynamic effects of intravenous paracetamol (acetaminophen) in patients with chronic liver disease undergoing liver transplantation: a pilot study

**DOI:** 10.1186/s13104-021-05749-8

**Published:** 2021-08-24

**Authors:** Laurence Weinberg, Elizabeth Chiam, Jadon Karp, Leonid Churilov, Rinaldo Bellomo

**Affiliations:** 1 Department of Anesthesia, Austin Health, Studley Rd, Heidelberg, VIC Australia; 2 Department of Surgery, The University of Melbourne, Austin Health, Victoria, Australia; 3 Monash School of Medicine, Monash University, Victoria, Australia; 4 Department of Medicine (Austin Health) and Melbourne Brain Centre at Royal Melbourne Hospital, Melbourne Medical School, Parkville, Australia; 5 Faculty of Medicine, Dentistry and Health Sciences, The University of Melbourne, Melbourne, Australia; 6 Department of Intensive Care, Austin Hospital, Heidelberg, VIC Australia; 7 Department of Critical Care, The Univesity of Melbourne, Victoria, Australia

**Keywords:** Paracetamol, Acetaminophen, Chronic liver disease, Liver transplantation, Hypotension, Hemodynamic effects

## Abstract

**Objective:**

We performed a single-center double-blinded, randomized trial to investigate the hemodynamic effects of IV paracetamol in patients with chronic liver disease (CLD) undergoing liver transplantation surgery. Patients with CLD are particularly susceptible to hemodynamic derangements given their low systemic vascular resistance state. Accordingly, hypotension is common in this setting. The hemodynamic effects of IV paracetamol in patients undergoing elective liver transplantation are unknown, therefore we evaluated whether the intraoperative administration of IV paracetamol in patients with chronic liver disease undergoing liver transplantation results in adverse hemodynamic effects. The primary end point was a change in systolic blood pressure 30-min after the preoperative infusion.

**Results:**

Twenty-four participants undergoing liver transplantation surgery were randomly assigned to receive a single bolus of IV paracetamol (1 g paracetamol + 3.91 g mannitol per 100 mL) (n = 12) or placebo (0.9% Saline 100 mL) (n = 12). All participants completed their study intervention, and there were no breaches or violations of the trial protocol. Baseline characteristics were similar in both groups. There were no significant differences regarding surgical duration, intraoperative use of fluids, and intraoperative noradrenaline use. After the administration of paracetamol there were no significant differences observed in blood pressure or other hemodynamic parameters when compared to placebo.

**Supplementary Information:**

The online version contains supplementary material available at 10.1186/s13104-021-05749-8.

## Introduction

Paracetamol (also known as acetaminophen) is a commonly used drug in the hospital setting to treat fever and mild to moderate pain, with its intravenous (IV) formulation routinely used in surgical and critically ill patients. Despite paracetamol’s reputation as the leading cause of drug-induced liver injury in the developed world, several studies support its safe use in the cirrhotic patient. When administered at the recommended daily dosage, paracetamol appears to be well-tolerated in all etiologies of liver disease including alcoholic cirrhosis and hepatitis C [1]. With its limited side effect profile, it is considered a safer and efficacious form of analgesia in surgical patients, compared with both non-steroidal anti-inflammatory drugs (NSAIDs) and opioids [1].

Recent clinical trials have shown that IV paracetamol may cause hypotension in healthy volunteers, the critically ill, and in cardiac surgery patients [2–5]. The hypotensive effects of IV paracetamol appear to be independent of the excipient mannitol (3.9 g), which is included as a stabilization agent in many of the commercially available IV paracetamol formulations. Whilst the osmotic diuretic effects of mannitol, even in these small doses, can theoretically cause transient hypotension, a recently published blinded, triple crossover, randomized trial of adult healthy volunteers receiving IV mannitol (3.9 g 100 mL), paracetamol formulation (3.9 g mannitol + 1 g paracetamol 100 ml) or 0.9% normal saline (100 ml), showed that the administration of mannitol did not result in any significant hemodynamic effects [6]. Whilst the hemodynamic effects of paracetamol have been studied in a variety of clinical settings, IV paracetamol has not been evaluated in patients with chronic liver disease (CLD). Such patients often have a reduced blood pressure and systemic vascular resistance index (SVRI), with an increased or unchanged cardiac index due to the hemodynamic alterations associated with a hyperdynamic circulatory state of end-stage liver disease [7]. Therefore, we conducted a double-blind, randomized controlled study to test the hypothesis that IV paracetamol lowers blood pressure in patients with chronic liver disease undergoing liver transplantation when compared to placebo.

## Main text

### Material and methods

Between November 2013 and March 2017, participants were identified from elective liver transplantation surgery waiting lists. Inclusion criteria included adult patients (age > 18 years), with chronic liver disease, undergoing elective liver transplantation surgery. Exclusion criteria included known allergic reaction to IV paracetamol, administration of paracetamol or NSAIDs within 24 h of surgery, caffeine consumption less than 10 h prior to surgery, pregnancy, severe chronic renal impairment (preoperative creatinine > 250 μmol/L) and morbid obesity (body mass index (BMI) > 35 kg/m^2^).

Sample size calculations were based on pilot data of preoperative blood pressure measurements in patients scheduled for liver transplantation at our institution. With an average blood pressure of 110 mmHg, and a standard deviation (SD) of 10 mmHg, to demonstrate a mean difference between the paracetamol group and control group of 10 mmHg, with a power value of 90%, a minimum of 11 participants would be required to be recruited into each group. To allow for violations or breaches in study protocol, 12 participants were recruited in each arm. A statistician generated a computerized sequence of 24 allocation codes, 12 for each group. An independent research nurse sealed the allocation codes into sequentially numbered opaque envelopes. The sequence was decoded after the data was analyzed.

On the day of surgery, an independent clinical pharmacist prepared one 100 mL IV infusion. All trial drug bottles contained paracetamol (1 g paracetamol + 3.91 g mannitol/100 mL) (Actavis Australia, The Rocks, NSW, Australia), or saline 0.9% (100 mL) (placebo) (Baxter Healthcare, Toongabbie, NSW, Australia) as a control. Intraoperative data collection was conducted after a standardized induction of anesthesia (fentanyl 1–3 μg/kg, propofol 1–2 mg/kg, and rocuronium 1 mg/kg). Anesthesia was maintained with isoflurane (0.5–1.5 MAC) in O_2_/air mixture (FiO_2_ = 0.5) and intravenous fentanyl (2–5 μg/kg/hr). All participants had a 18 cm 18G arterial line (Leardale, Vygon, UK) inserted into their non-dominant brachial artery and femoral artery. A continuous cardiac output pulmonary artery catheter (CCombo, Edwards Lifesciences, North Ride, NSW) was inserted via the right internal jugular vein. After the insertion of invasive lines, participants were placed in 15-degree head-up position, with their head resting on a pillow for comfort. IV fluids, or any other medications were avoided unless clinically required.

Participants were given a 15-min stabilization period before baseline hemodynamic measurements were recorded. After the stabilization period, the study drug was infused at room temperature over 15 min. The primary end point was an absolute change in systolic blood pressure (SBP) from the baseline to 30 min. We measured changes in mean and systolic blood pressures, diastolic blood pressures (DBP), cardiac index, pulmonary artery pressures, systemic vascular resistance index (SVRI) and heart rate (HR). Pulmonary artery pressure was measured directly from the pulmonary artery catheter. Cardiac index was measured continuously via the continuous cardiac output pulmonary artery catheter.

Measurements were recorded at 5, 8, 15 and 30 min after the start of the preoperative infusion and 15 and 30 min after the start of the postoperative infusions, with hourly measurements for six consecutive hours. Other data collected included baseline patient characteristics, intraoperative use of fluids and vasoactive medications, cardiopulmonary bypass time, aortic cross-clamp time, surgery duration, intraoperative use of fluid and vasoactive medications, ventilator times, and length of ICU and hospital stay.

Statistical analysis was performed using commercial statistical software STATA/IC v.13 with a p-value of 0.05 to indicate statistical significance. The primary end point (absolute change in SBP from the baseline to 30 min) was analyzed using an ANCOVA model with the SBP value at 30 min as an outcome, the treatment group as a factor, and the baseline SBP value as a treatment covariate. All other end points were analyzed using random effect generalized least squares regression modelling due to the repeated measures nature of the data. The study adheres to the CONSORT Guidelines for reporting randomized trials [8].

## Results

**Fig. 1:**
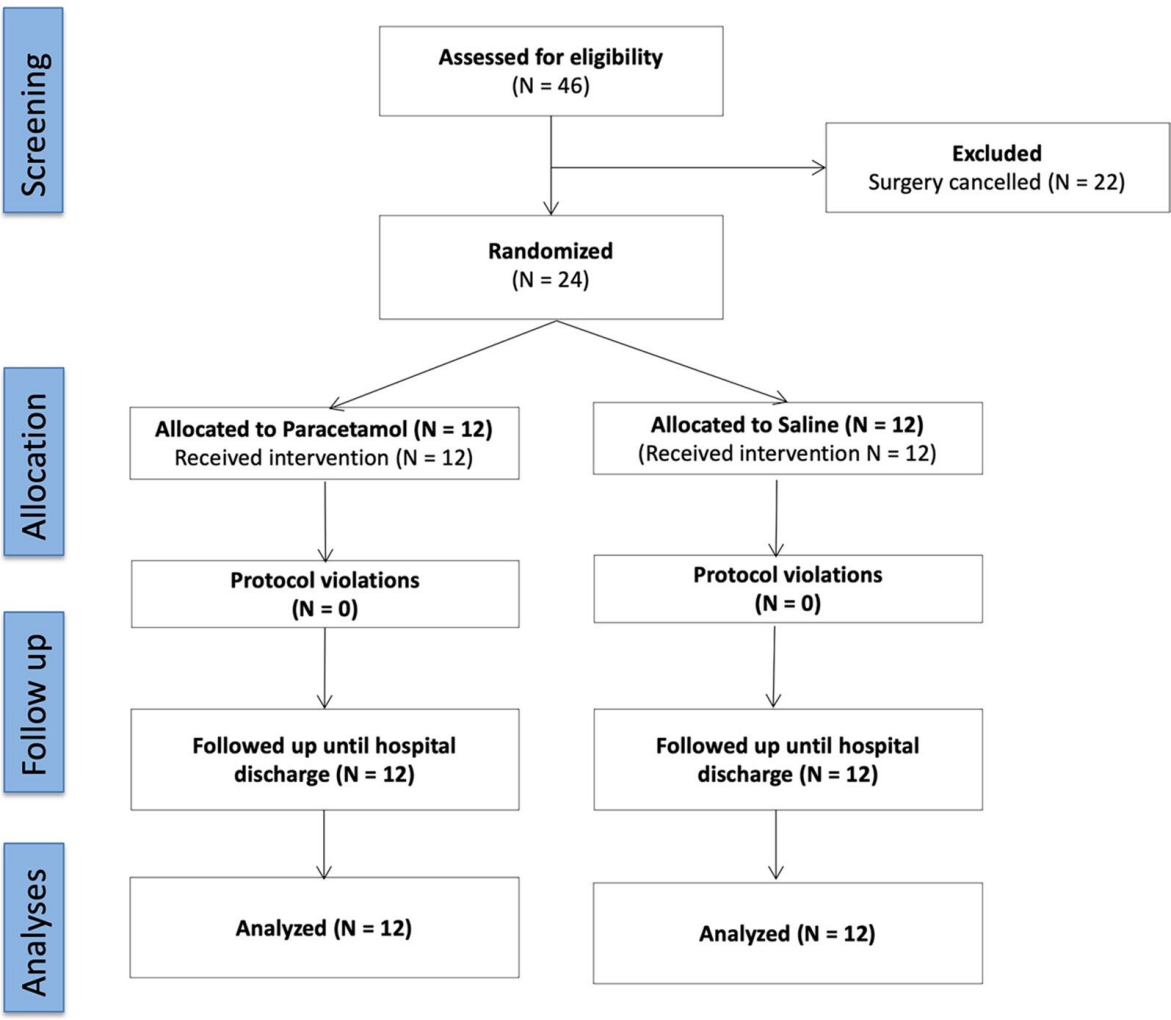
CONSORT diagram

The study CONSORT diagram is presented in Fig. 1. Baseline characteristics, including age, gender, and body mass index were similar in both groups and summarized in Table 1. The mean (standard deviation) Model for End-stage Liver Disease Score was 20.1 (6.8) in the paracetamol group and 14.9 (4.7) in the control group. There were no significant differences observed regarding surgical duration, intraoperative use of fluids and intraoperative noradrenaline use.

**Table 1:** Summary of patient demographics

Preoperative characteristics	Paracetamol (N = 12)	Saline (N = 12)
Age (years)	56.9 (8.5)	59.0 (7.0)
Male gender	5 (42%)	9 (75%)
Weight (kg)	83.5 (36.8)	96.1 (41.3)
Height (cm)	167.8 ± 9.3	171.3 ± 10.2
Body mass index (kg/m^2^)	21.6 (11.3)	23.2 (10.4)
Model for End-stage Liver Disease Score	20.1 (6.8)	14.9 (4.7)

Indication for liver transplant	Paracetamol (N = 12)	Saline (N = 12)
Hepatitis C	1 (8%)	4 (33%)
Hepatitis C + Hepatocellular carcinoma	5 (42%)	3 (25%)
Hepatitis B	0 (0%)	2 (17%)
Hepatitis B + Hepatocellular carcinoma	1 (8%)	0 (0%)
Primary sclerosing cholangitis	2 (17%)	1 (8%)
Non-cirrhotic portal hypertension	2 (17%)	1 (8%)
Alcohol induced liver failure	1 (8%)	0 (0%)
Non-alcoholic steatohepatitis	0 (0%)	1 (8%)

Intraoperative variables	Paracetamol (N = 12)	Saline (N = 12)	P-value
Surgery duration (mins)	475.7 (105.0)	446.5 (47.9)	0.4
Intraoperative fluid (mL)	6387 (3273)	6234 (2771)	1.0
Intraoperative noradrenaline (µg)	1775 (1932)	1868 (1407)	0.7

Data is mean (standard deviation) or number (proportion in percentage)

**Fig. 2:**
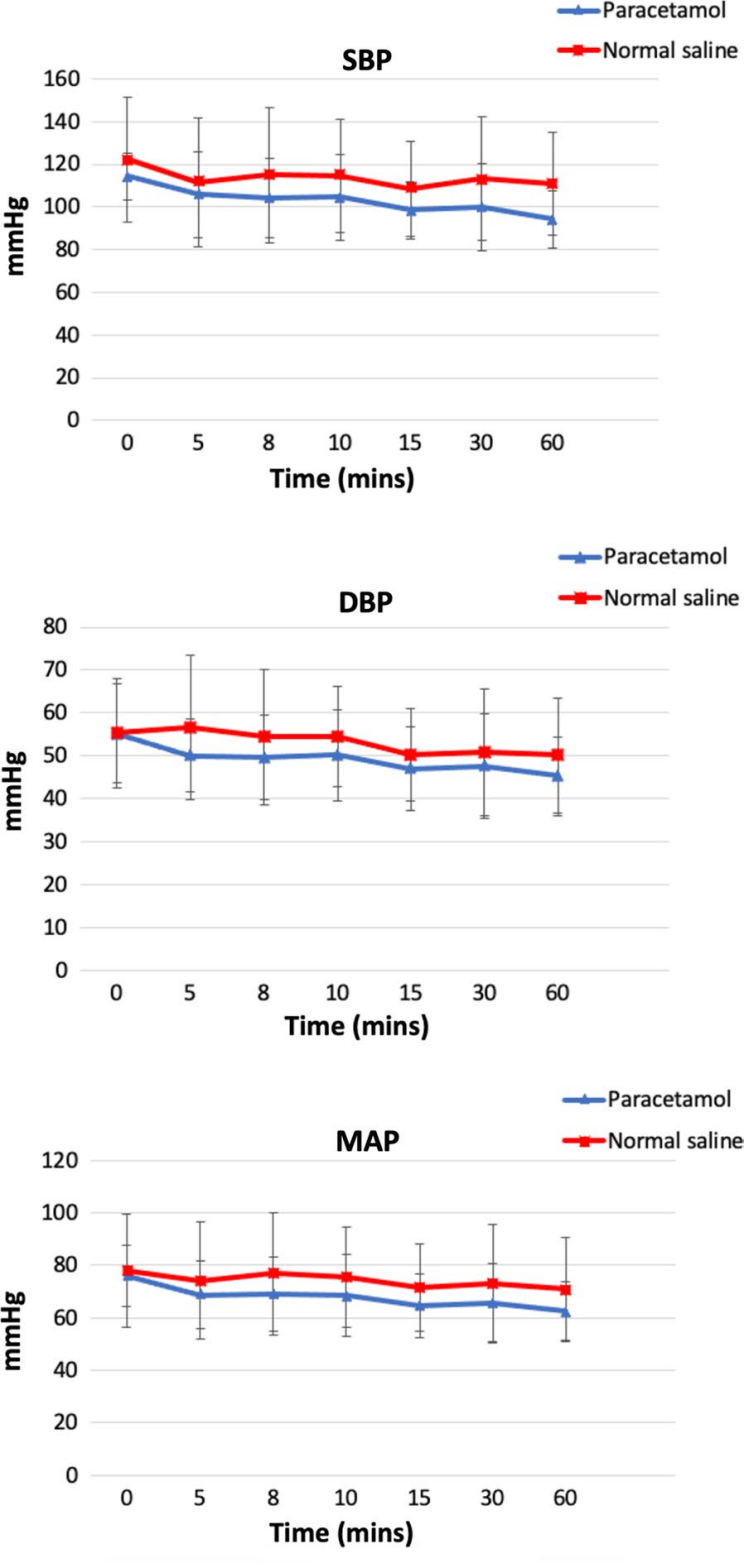
Changes in systolic (SBP), diastolic (DBP) and mean (MAP) arterial pressure after paracetamol administration. Values are mean (standard deviation)

Changes in SBP, DBP and MAP after the paracetamol or Saline infusions from baseline to the 60-min end point are summarized in Fig. 2. Changes in hemodynamic variables from baseline to the 60 min after the preoperative infusions are summarized in the Additional file 1: Table S1.

Fifteen minutes after the start of infusion, SBP decreased from 114 (11) mmHg to a nadir of 98 (13) mmHg in the paracetamol group, representing a decrease of 14.0% from the baseline. Conversely, 15 min after infusion the Saline group, SBP decreased from 122 (29) mmHg to 109 (22) mmHg. This resulted in a decrease of 10.66% from the baseline. The p-value adjusted for treatment-by-time interaction was 0.57 indicating no statistical significance between the two groups. Over the 60-min study period, there was no statistical or clinically significant decreases in DBP observed between the two groups. DBP dropped from 55 (12) mmHg to a nadir of 45 (9) mmHg) in the paracetamol group, representing a 18.9% decrease from the baseline 60 min after infusion. Saline had a nadir 9.1% decrease in DBP from the baseline 30 min after infusion. The p-value adjusted for treatment-by-time interaction was listed as 0.54. Finally, paracetamol also decreased MAP from 76 (12) mmHg to a nadir of 63 (11) mmHg over the 60-min study period, with a decrease of 17.11% from baseline. This was seen to a lesser extent in the Saline group with an overall decrease of 8.97% from baseline 60 min after infusion. The p-value adjusted for treatment-by-time interaction was 0.61.

Analyses of SVRI, cardiac index, pulmonary artery pressures, and HR showed no statistical significance in their interaction between the treatments over time. There were no significant changes in blood pressure between the groups after adjustments for age, BMI, gender and Model for End-stage Liver Disease Score.

## Discussion

To date this is the first study providing plausible physiological evidence that intraoperative administration of 1 g of IV paracetamol in patients with CLD undergoing liver transplantation caused no changes in blood pressure when compared with a placebo.

The use of paracetamol in the context of liver disease is often avoided due to concerns regarding paracetamol-induced liver toxicity [9]. NAPQI or N-acetyl-p-benzoquinone imine is a hepatotoxic metabolite produced during the metabolism of paracetamol. It is normally produced in small amounts and then immediately detoxified in the liver. There are two important factors involved in the production of NAPQI: insufficient glutathione levels and increased activity of the CYP2E1 pathway. Interestingly, in patients with cirrhosis, glutathione levels are adequate and the CYP pathway activity is not upregulated upon administration of the maximum 4 g per day dosage [10]. Thus, use of the recommended dosages of paracetamol in this population should be considered safe.

The hemodynamic effects of IV paracetamol have largely centered around results from studies conducted in critically ill patients. Whilst these studies suggest IV paracetamol may have the propensity to cause hypotension, many are limited by small patient numbers, retrospective design, lack of randomization and blinding or being anecdotal in nature. In addition, frequent use of vasopressors in this patient cohort may confound the results by masking the effect of the paracetamol-induced hypotension [3, 4, 11, 12]. The clinical significance of the proposed hemodynamic effects of IV paracetamol in other patient subgroups is yet to be established. Needleman found that a rapid infusion of IV paracetamol in ambulatory surgical patients (ASA Class I–III) produced a statistically significant decrease in blood pressure over a short 5-min observation period. These results were not supported by a clinically significant change in blood pressure [13]. Moreover, the study did not provide any information on the baseline hemodynamics nor followed up on hemodynamic changes after the short observation period. It is therefore possible that the short duration of the observation period may have resulted in the inability to identify and report the subsequent occurrence of any hypotensive episodes.

Krajcova et al. found that repeated administrations of IV paracetamol reduced MAP in 6 critically ill patients [5]. This was accompanied by a reduction in SVRI and cardiac index. Similar findings were replicated in a randomized, blinded, controlled crossover trial where IV paracetamol caused a significant decrease in both blood pressure and SVRI [6]. The latter study showed no changes in cardiac index upon infusion of paracetamol; however, both studies suggested that vasodilation may be a mechanism of the IV paracetamol-induced hypotension. Chiam et al. postulated that the hemodynamic effects of IV paracetamol may be of a different magnitude and intensity in other patients, specifically those vulnerable to hypotension and lowered SVR states e.g., in septic shock [13].

## Conclusion

In a randomized blinded trial comparing the hemodynamic effects of IV paracetamol and placebo (saline) in patients with chronic liver disease undergoing liver transplantation, we found that the intraoperatively administration of paracetamol caused no significant changes in blood pressure when compared to placebo. Our findings support the therapeutic benefits of the safe use of the recommended dosage of IV paracetamol within this patient cohort.

## Limitations

We provided insights on the hemodynamic effects of paracetamol on a very small cohort of 24 liver transplant recipients. We cannot extrapolate our paracetamol infusion results to different combinations of rate, duration or route. However, the dose and speed of administration in our patients reflect clinical practice and recommendations. The findings with IV paracetamol are likely not relevant to oral or per-rectum paracetamol, where the absorption is slower and any hemodynamic effects that might exist are attenuated. We only explored the hemodynamic effects of IV paracetamol in patients with CLD. Therefore, we did not measure the biochemical effects of paracetamol on liver function tests, nor did we examine the effects of paracetamol and its metabolite NAPQI on liver function or graft outcomes. Moreover, the study was not intended to explore any clinical outcomes.

## Supplementary Information

**Additional file 1: Table S1**. Hemodynamic changes in patients with chronic liver disease undergoing liver transplantation after IV paracetamol administration. Values are mean values (standard deviation) with 95% confidence intervals (95% CI). (SBP, systolic blood pressure; DBP, diastolic blood pressure; MAP, mean arterial pressure; sPAP, systolic pulmonary artery pressure; dPAP, diastolic pulmonary artery pressure; mPAP, mean pulmonary artery pressure; CVP, central venous pressure; HR, heart rate; CI, cardiac index; SVRI, systemic vascular resistance index.

## Data Availability

All data produced or analysed during the current study are included in this article. The original datasets and other miscellaneous materials related to the trial are available upon reasonable request to the corresponding author.

## Abbreviations

CLD: Chronic liver disease
SVR: Systemic vascular resistance
IV: Intravenous
NSAID: Non-steroidal anti-inflammatory drugs
SVRI: Systemic vascular resistance index
BMI: Body mass index
SD: Standard deviation
MAC: Minimum alveolar concentration
SBP: Systolic blood pressure
DBP: Diastolic blood pressure
HR: Heart rate
MAP: Mean arterial pressure
sPAP: Systolic pulmonary artery pressure
dPAP: Diastolic pulmonary artery pressure
mPAP: Mean pulmonary artery pressure
CVP: Central venous pressure
CI: Cardiac index
ASA: American Society of Anesthesiologists
