# Supplementary material for: The hemodynamic effects of intravenous paracetamol (acetaminophen) in patients with chronic liver disease undergoing liver transplantation: a pilot study
Source: BMC Res Notes. 2021 Aug 24;14:325. doi: 10.1186/s13104-021-05749-8 (PMC8383248; doi:10.1186/s13104-021-05749-8)
Supplement: Supplementary file 1 — Additional file 1: Table S1. Hemodynamic changes in patients with chronic liver disease undergoing liver transplantation after IV paracetamol administration. Values are mean values (standard deviation) with 95% confidence intervals (95% CI). (SBP, systolic blood pressure; DBP, diastolic blood pressure; MAP, mean arterial pressure; sPAP, systolic pulmonary artery pressure; dPAP, diastolic pulmonary artery pressure; mPAP, mean pulmonary artery pressure; CVP, central venous pressure; HR, heart rate; CI, cardiac index; SVRI, systemic vascular resistance index. [file 13104_2021_5749_MOESM1_ESM.docx]

**Table S1: Hemodynamic changes in patients with chronic liver disease undergoing liver transplantation after IV paracetamol administration.** Values are mean values (standard deviation) with 95% confidence intervals (95% CI). (SBP, systolic blood pressure; DBP, diastolic blood pressure; MAP, mean arterial pressure; sPAP, systolic pulmonary artery pressure; dPAP, diastolic pulmonary artery pressure; mPAP, mean pulmonary artery pressure; CVP, central venous pressure; HR, heart rate; CI, cardiac index; SVRI, systemic vascular resistance index.

|  |  | **Baseline** | **5 mins** | **8 mins** | **10 mins** | **15 mins** | **30 mins** | **60 mins** | **Mean difference** | **95% CI** | ***p* value (treatment)** | ***p* value (treatment-by-time interaction)** |
| --- | --- | --- | --- | --- | --- | --- | --- | --- | --- | --- | --- | --- |
| SBP (mmHg) | **Paracetamol** | 114 (11) | 106 (20) | 104 (18) | 105 (20) | 98 (13) | 100 (20) | 94 (13) | -10.80 | -25 to 4 | 0.16 | 0.57 |
|  | **Saline** | 122 (29) | 112 (30) | 115 (32) | 115 (27) | 109 (22) | 113 (29) | 111 (24) |  |  |  |  |
| DBP (mmHg) | **Paracetamol** | 55 (12) | 50 (9) | 50 (10) | 50 (11) | 47 (10) | 48 (12) | 45 (9) | -3.85 | -11 to 4 | 0.32 | 0.54 |
|  | **Saline** | 55 (13) | 57 (17) | 54 (16) | 55 (12) | 50 (11) | 51 (15) | 50 (13) |  |  |  |  |
| MAP (mmHg) | **Paracetamol** | 76 (12) | 69 (13) | 69 (14) | 68 (16) | 65 (12) | 66 (15) | 63 (11) | -6.43 | -17 to 5 | 0.96 | 0.61 |
|  | **Saline** | 78 (21) | 74 (22) | 77 (23) | 75 (19) | 72 (16) | 73 (22) | 71 (20) |  |  |  |  |
| sPAP (mmHg) | **Paracetamol** | 31 (11) | 41 (32) | 31 (11) | 32 (12) | 40 (28) | 28 (11) | 29 (10) | 0.07 | -10 to 10 | 0.99 | 0.60 |
|  | **Saline** | 34 (13) | 32 (14) | 34 (13) | 34 (12) | 34 (12) | 33 (15) | 31 (13) |  |  |  |  |
| dPAP (mmHg) | **Paracetamol** | 14 (4) | 19 (17) | 13 (5) | 14 (5) | 15 (14) | 12 (4) | 11 (4) | 0.86 | -3 to 5 | 0.70 | 0.45 |
|  | **Saline** | 15 (7) | 13 (8) | 13 (6) | 13 (5) | 13 (5) | 13 (5) | 12 (4) |  |  |  |  |
| mPAP (mmHg) | **Paracetamol** | 21 (7) | 28 (21) | 21 (7) | 22 (7) | 25 (17) | 19 (7) | 19 (7) | 0.76 | -2 to 4 | 0.59 | 0.69 |
|  | **Saline** | 21 (8) | 20 (9) | 20 (9) | 22 (7) | 22 (7) | 21 (9) | 20 (7) |  |  |  |  |
| CVP (mmHg) | **Paracetamol** | 11 (6) | 11 (7) | 10 (5) | 11 (6) | 10 (6) | 9 (5) | 8 (5) | 0.02 | -3 to 3 | 0.99 | 0.78 |
|  | **Saline** | 11 (4) | 10 (4) | 10 (3) | 10 (3) | 10 (3) | 9 (4) | 9 (4) |  |  |  |  |
| HR (beats/min) | **Paracetamol** | 82 (11) | 80 (14) | 79 (12) | 78 (13) | 76 (13) | 77 (15) | 77 (13) | 0.80 | -8 to 10 | 0.87 | 0.83 |
|  | **Saline** | 79 (15) | 77 (12) | 77 (11) | 77 (12) | 78 (13) | 77 (13) | 79 (13) |  |  |  |  |
| CI (L min/m^2^) | **Paracetamol** | 3.90 (0.87) | 3.82 (0.93) | 3.92 (0.97) | 4.11 (0.89) | 4.00 (0.89) | 4.16 (1.11) | 4.22 (0.97) | -0.02 | -0.80 to 0.76 | 0.96 | 0.79 |
|  | **Saline** | 4.10 (1.26) | 3.97 (1.33) | 3.95 (1.17) | 3.96 (1.17) | 4.01 (0.92) | 4.13 (1.11) | 4.14 (1.25) |  |  |  |  |
| SVRI (dynes/sec/cm^-5^/m^2^) | **Paracetamol** | 1429 (561) | 1275 (476) | 1305 (526) | 1181 (389) | 1162 (428) | 1135 (347) | 1078 (321) | -178 | -582 to 226 | 0.39 | 0.75 |
|  | **Saline** | 1436 (700) | 1441 (679) | 1504 (763) | 1462 (661) | 1311 (569) | 1356 (677) | 1304 (564) |  |  |  |  |
